# Adoption of Major Housing Adaptation Policy Innovation for Older Adults by Provincial Governments in China: The Case of Existing Multifamily Dwelling Elevator Retrofit Projects

**DOI:** 10.3390/ijerph19106124

**Published:** 2022-05-18

**Authors:** Yongqiang Chu, Shuguang Shen

**Affiliations:** 1 Institute of Talent Assessment and Development for the Guangdong-Hong Kong-Macau Greater Bay Area, Guangdong University of Finance & Economics, Guangzhou 510320, China; 2 Social Policy Research Center, Guangdong University of Finance & Economics, Guangzhou 510320, China; 3 Lingnan (University) College, Sun Yat-sen University, Guangzhou 510320, China; ssg84114060@163.com

**Keywords:** aging in place, housing adaptation, elevator retrofit, policy innovation, piecewise constant exponential (PCE) model

## Abstract

(1) Background: The housing environment is crucial to the health of older Chinese people and is becoming an urgent policy initiative. This study explores factors that facilitate or impede the adoption of policy innovation on major housing adaptation (HA) by Chinese provincial governments using the framework of policy innovation and diffusion theory. (2) Methods: This study constructs an event history dataset on HA policy related to elevator retrofitting in existing multifamily dwellings in China; the lack of elevators constitutes an insurmountable barrier in older adults’ daily lives in China. The hypotheses were tested by using a traditional event history analysis (EHA) model and a piecewise constant exponential (PCE) model, which is a modified EHA model. The dataset was summarized as “province-year” event history data on 30 Chinese provinces from 2008 to 2019. (3) Results: In addition to internal determinants (e.g., population aging level and financial dependency), diffusion mechanisms can significantly facilitate or impede the adoption of major HA policy innovation by provincial governments. Policy adoption by neighboring governments helps facilitate policy adoption by nonadopters, but policy adoption by subordinate city governments impedes provincial governments’ adoption of major HA policy innovation. (4) Conclusions: This study concludes that provincial governments’ adoption of major HA policy innovation should be given a higher policy priority. The central government can promote provincial governments’ adoption of major HA that primarily benefits older adults by using fiscal transfer payments and enhancing the legitimacy of such policy.

## 1. Introduction

The housing environment is critical to the health of older adults living in a community. With the decline in physical function, older adults living in inaccessible housing, i.e., those with physical barriers at home and in their immediate surroundings, will encounter more environmental barriers, home hazards, fall-related risks, and limitations to their mobility when they age at home [1,2,3]. Housing adaptation (HA) refers to modifications to the permanent physical features in the indoor and immediate outdoor environment. These modifications range from minor adaptations (grab bars, wheelchair accommodation, etc.) to major adaptations (elevator installation, auto door installation, etc.) [4]. HA is recognized as an effective intervention to improve housing accessibility and maintain and restore the independent living of older adults [5]. Among some aging nations, HA has been incorporated into their social policy frameworks to develop HA services for older adults and the disabled at the local level [6,7,8,9]. 

China is experiencing rapid population aging and faces increasing pressure to provide accessible housing and satisfy the changing needs of its aging population. Between 2019 and 2030, the share of the population aged 65 years and over is projected to increase from 11.5% to 16.9% of China’s total population [10]. The demand for accessible housing to satisfy the changing needs of older adults is simultaneously growing and has become a new policy issue for Chinese governments at multiple levels. Older adults living in multifamily dwellings in deteriorating urban communities suffer the most serious and urgent housing accessibility problems, especially environmental barriers concerning stair features. For example, many of these older adults state that a lack of access to an elevator constitutes an insurmountable barrier in their daily lives [4,6,7], potentially leaves them trapped in their homes, and accelerates their isolation from the community. However, such major housing accessibility problems caused by the lack of an elevator may be more challenging to address, not only because an elevator (compared to more common modifications, such as grab bars) is financially out of reach but also because efforts to install an elevator may encounter the “not in my back yard” phenomenon and trigger neighborhood conflicts [6,7]. The engagement of multiple governments in this domain is very important. In China, as in most aging countries, mainly governments at the city or municipal level are responsible for providing HA services to older residents [6,7,8]. However, large gaps have appeared in the delivery of major HAs to older adults among cities or municipalities in the provinces. Only cities with more financial resources, higher governmental capacity, and an increasing population are likely to institute policy innovations addressing the most urgent major HA services for older adults [6,7,11]. Improving access by older adults who live in these kinds of cities to major HA services should be given a higher policy priority, and provincial-level policy planning and coordination are relevant to improving such access. However, the discussion of the adoption of major HA policy innovations by provincial governments has been very limited in the academic world. To bridge this research gap, this study attempts to explore what factors facilitate or impede Chinese provincial governments’ adoption of major HA policy innovations that benefit primarily older adults. To achieve its aim, this study uses the framework of policy innovation and diffusion theory based on the case of existing multifamily dwelling elevator retrofit projects in China.

## 2. The Policy Context of Existing Multifamily Dwelling Elevator Retrofit Projects in China

**Figure 1:**
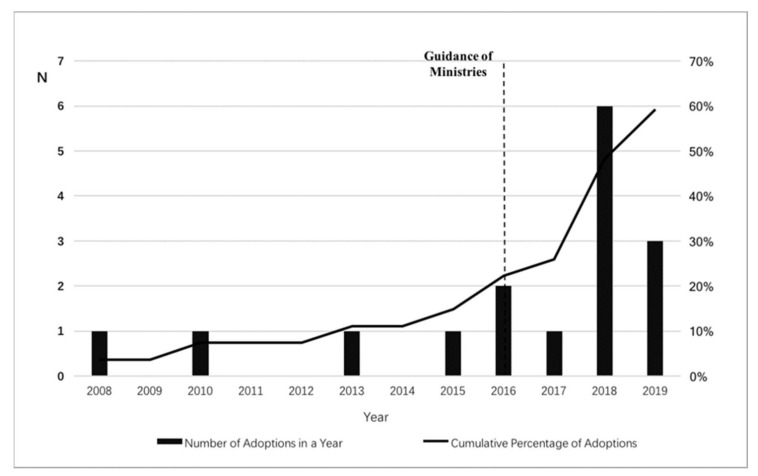
The adoption of the EMDER policy by Chinese provincial governments (2008–2019).

Most of the multifamily dwellings discussed in this study were built during the 1990s when China faced severe housing shortages, and governments at multiple levels built many multifamily dwellings without elevators. At the time, these dwellings were designed primarily to accommodate the needs of young adults and did not consider the needs of older adults. As people age in these buildings, it becomes harder for them to walk up and down stairs; the lack of elevators has seriously affected these residents’ quality of life and fostered isolation from the community. In recent years, there has been a broad public debate on elevator retrofitting in these older and deteriorating multifamily dwellings. In practice, elevator retrofitting in existing multifamily dwellings is complex and is thus beyond the traditional boundaries of many government departments, requires coordinating conflicts of interest among residents, and easily triggers social risks. Even so, there is an emerging consensus regarding the innovative policies and programs needed to address this pressing social problem. In some provinces, provincial governments take the leading role in adopting and implementing major HA policy innovations (elevator retrofitting) for older adults in China. There is a clear province-led policy to encourage and balance the subordinate cities to provide major HA services to these older residents. For example, the Sichuan provincial government has adopted a new policy, that is, the Existing Multifamily Dwellings Elevator Retrofit (EMDER) policy, to guide and promote the implementation of policy innovation on major HA services for older adults throughout the province. This policy clarifies some important rules and financing arrangements for major HA services (elevator retrofitting) that benefit primarily older adults and the disabled. First, qualifications for existing multifamily dwellings can be adapted. The application and acceptance of elevator retrofit projects for existing multifamily dwellings need to meet an important premise, that is, to obtain the consent of more than two-thirds of the homeowners of a building. Second, the financing system for elevator retrofitting in existing multifamily dwellings includes out-of-pocket payments by homeowners, housing provident funds, and subsidies from provincial or city governments. Specifically, elevator retrofitting should be funded by the homeowners themselves first, and residents are responsible for contributing a proportion of the cost corresponding to the floor they live on. Then, the Housing Provident Fund can be a source of funding to broaden the funding channels for this HA service. Finally, provincial governments provide financial support to poorer and smaller cities through special transfer payments, such as awards and subsidies, to promote the adoption and implementation of this elevator retrofitting work at the city level. In 2016, China’s first national guidance on the Livable Community Environment for Older Adults was released. This national guidance encourages and provides support for local governments to experiment with policy innovations on HAs for older adults. By 2019, almost half of the provinces in China had adopted the EMDER policy. Figure 1 presents the adoption of the EMDER policy at the provincial level in China. In this study, the major HA policy refers to the Existing Multifamily Dwellings Elevator Retrofit (EMDER) policy in China.

## 3. Theoretical Framework

Policy innovation and diffusion theory was developed to answer such questions. According to Walker’s classical definition, state government innovation can be defined as a “program or policy which is new to [the state] adopting it”, and the central research question about state innovation is, what causes a government to adopt a new program or policy? [12]. This theory has been identified as a useful framework for understanding the adoption of age-friendly policy innovations by governments in verified country contexts [6,7]. In general, existing studies have developed two principal types of models that explain why local governments adopt a new policy: internal determinant and diffusion models [12,13,14]. Internal determinant models refer to the factors driving a government to adopt new policies; these factors are political, economic, or social characteristics (such as market needs and local residents’ demands) internal to the government’s jurisdiction and the local policy environment [15]. In contrast, diffusion models posit that policy adoption by a government is also influenced by other governments’ actions, which are inherently intergovernmental interactions. These two distinctive models imply different understandings of the factors, which facilitated or impeded the adoption of the EMDER policy by provincial governments in China.

### 3.1. Internal Determinants

The policy innovation diffusion model argues that local policy innovation can be influenced by internal factors, which constitute the local policy environment [12]. The internal factors examined in the study include issue salience and fiscal dependency, which could condition the adoption of major HA policy innovations for older adults at the local level [15,16].

#### 3.1.1. Issue Salience

A salient issue is defined usually as “one which is important to a sizeable portion of the population either because it directly affects their well-being or touches areas about which they are vicariously concerned” [15]. That is, the larger the affected population in a jurisdiction is, the more salient the issue will be in this jurisdiction. Highly salient issues may give policymakers a sense of urgency and pressure by raising the public awareness of more people. This kind of policy environment will catalyze the responsive policy innovation of local governments on this issue [17]. Some existing studies have found that *issue salience*, measured as the size of the affected group in a jurisdiction, can significantly facilitate the adoption of age-friendly policy innovation by city governments [6]. Specific to the study, the households that live in existing multifamily dwellings without elevators more urgently advocate for changes to their physical environment because households are more aware of the physical barriers in their buildings keenly when household members age. Such an urgent social demand spreads through the internet, news media, etc., thus causing widespread public concern and appeal for government policy innovation. In other words, jurisdictions with a higher proportion of households affected are more likely to adopt policy innovations to satisfy the demand of the affected group. Thus, this study develops the following hypothesis:

**Hypothesis** **1.** *A provincial government with a higher proportion of affected households is more likely to adopt the EMDER policy.*

#### 3.1.2. Fiscal Dependency

China’s Tax-Sharing System Reform, established in 1994, shapes the vertical intergovernmental fiscal relationship, which could significantly influence the policy decision-making of local governments [18,19]. Under this system, the central government obtains a larger share of tax revenue, but local governments are still responsible for many fiscal expenditures. In some places, local revenue alone cannot meet the broad expenditure responsibilities of local governments. In this case, local governments may compete for various intergovernmental transfer payments from the central government to meet such fiscal gaps. These transfer payments have become a significant component of local expenditure and are particularly important to local governments with poor tax bases. Thus, some local governments have an inherent incentive to follow the policy goals of superior governments. Moreover, under this system, superior governments usually have a greater capability to intervene in local government policymaking. The central government can also use earmarked transfers to stimulate local governments’ policy innovation to comply with the central government’s policy goals. Specific to the study, HA for older adults was recognized as a social welfare expenditure. Thus, local governments generally lack the incentive to adopt policy innovation and expand social investment in this domain. However, those who depend heavily on transfer payments from superior governments have stronger incentives to respond to central government initiatives. We can expect that fiscal dependency on the superior government will play an important role. Thus, this study develops the following hypothesis:

**Hypothesis** **2.** *A provincial government with a greater fiscal dependency on the central government is more likely to adopt the EMDER policy.*

### 3.2. Diffusion Mechanisms

Diffusion mechanisms highlight the relevance of intergovernmental interactions on the policy decision making of local governments. China has three main administrative levels: central, provincial, and prefecture-level cities. Building on the theoretical advancements in the literature [13,18,20], this study examines three types of policy diffusion mechanisms: (1) top-down diffusion from the central government, (2) bottom-up diffusion from subordinate city governments, and (3) horizontal diffusion from neighboring governments.

#### 3.2.1. Top-Down Diffusion from the Central Government

In China, the central government plays an active role in adopting many government innovations at the local level [18,21]. In China’s vertical hierarchy, although provincial governments have attained a level of autonomy in recent decades, the central government can still influence the policymaking of provincial governments [18,22]. The central government’s policy objectives play an important role in setting policy priorities for local governments. Recognition and encouragement from the central government can send a positive political signal, which can legitimize the policy innovation of local governments [18]. The National Commission on Aging (NCA) at the central level plays a leading role in promoting the transformation of an aging society and is primarily responsible for policy advocacy and the coordination and promotion of relevant departments to jointly serve older adults. In 2016, NCA worked with 24 other central ministries to jointly issue China’s first national-level official guidance document, which is titled ‘Guidelines on Promoting the Construction of a Livable Environment for Older People’, to encourage local governments to experiment with policy innovations to achieve major HA for older people. Considering this clear policy signal from the central government, this study develops a top-down diffusion hypothesis:

**Hypothesis** **3.** *The encouragement of HA for older adults from the central government will increase the likelihood that a province will adopt the EMDER policy (central signal hypothesis).*

#### 3.2.2. Bottom-Up Diffusion from Subordinate City Governments

China is a vast country with multiple levels of government: province, prefecture city, county, and township. There is also extensive interaction between provincial governments and subordinate city governments, and the policy practice of the subordinate government may have multiple mechanisms to influence the policy adoption of the superior government. Specific to the study, the bottom-up influence from subordinate city governments may have two opposing effects: the positive snowball effect and the negative pressure valve effect [13,20]. First, with China’s decentralization reform after 1978, much of the social and economic policy related to social welfare was devolved to local governments, thus greatly expanding the policy autonomy of local governments, and providing them with an increasingly important role in improving the well-being of Chinese older adults [23,24,25,26]. The prefectural-level governments of China are responsible primarily for social and health service delivery for older adults and are more innovative in the aging policy domain than ever. In this case, the city government essentially becomes the policy laboratory for provincial governments to observe and evaluate policy innovation [27]. Specifically, provincial government officials will look to cities for policy ideas and solutions that these officials can advance at the provincial level. In other words, city governments can inform superior provincial policymakers about the success of varied policy innovations [14]. Thus, the increasing adoption of one policy innovation at the city level in a province may signal the policy innovation’s political viability, stability, and effectiveness; consequently, the policy innovation is more likely to be recognized and further adopted by provincial governments. Therefore, there may be a “positive snowball effect” in bottom-up diffusion from subordinate city governments. Thus, this study proposes the following hypothesis:

**Hypothesis** **4** **(Snowball** **Effect** **Hypothesis).** *The increasing adoption of the EMDER policy at the city level increases the likelihood of policy adoption at the provincial level (city adoption).*

Second, there may be an opposing “pressure valve effect” between provincial and municipal governments, in that policy adoptions by subordinate city governments may have completely opposite effects on the policy decisions of superior provincial governments in China. Highly salient issues may give policymakers a sense of urgency and pressure by raising public awareness, thereby leading more people to encourage local governments to adopt policy innovation [17]. According to Shipan and Volden’s argument, the policy problem to be addressed may become less salient and state government policymakers may feel less pressure if the issue has been addressed in the lower jurisdictions that most demanded policy change [13]. Specific to the study, in China, elevator retrofitting for older adults in existing multifamily dwellings has attracted extensive public attention and brought significant public pressure on governments at multiple levels. In China’s multilevel system, local governments at the prefectural level rather than the provincial level take primary responsibility for housing provision and adaptation for citizens, and policy change is most in demand at this level. The policy problem to be addressed at the subordinate city level will ease public pressure on provincial governments. Thus, increasing policy adoption at the city level may be a disincentive for provincial governments to address the same problem on a larger scale. Thus, this study proposes the following hypothesis:

**Hypothesis** **5** **(Pressure** **Value** **Effect** **Hypothesis).** *The increasing adoption of the EMDER policy by city governments decreases the likelihood of provincial governments’ policy adoption (city adoption).*

#### 3.2.3. Horizontal Diffusion from Neighboring Governments

Horizontal diffusion refers to peer pressure from neighboring governments; this pressure could also affect the decisions of local governments to adopt policy innovations for legitimacy reasons [28], especially in social policy. The legitimization of a policy refers to the perception that specific aspects of the policy are “desirable, proper, or appropriate within some socially constructed system of norms, values, beliefs and definitions” [28]. With more adopters, innovation gains more legitimacy, and the resulting pressure on nonadopters increases. Since population aging has become a widely noted policy issue in China, the adoption of an expanding housing innovation that primarily benefits older adults can provide legitimacy to provincial governments and cause an institutional “bandwagon effect”. Hence, this study proposes the following hypothesis:

**Hypothesis** **6.** *The likelihood of a province adopting the EMDER policy increases when the same policy is broadly adopted by other provinces throughout the country (neighboring adoption hypothesis).*

## 4. Research Methods

### 4.1. Data

The provinces observed in the study include 30 provinces in China. To ensure the consistency of the results, four municipalities directly under the central government were not included in this analysis because these municipalities had no subordinate cities. The time frame is from 2008, when Guangdong province established China’s first provincial-level EMDER policy, to 2019, when almost half of the provinces adopted the policy.

Collecting data on the events of EMDER policy adoption is time consuming. The study constructs an event history dataset on the adoption of the EMDER policy by provincial governments in China between 2008 and 2019. Without a public database of provincial government policy documents, the EMDER policy documents and related information had to be obtained from multiple sources. First, information on the year of the EMDER policy was recorded from the official notification for the establishment of the policy in each province. Second, information on independent variables was collected from the China Population Census (2010) and China Statistical Yearbook (2008–2019) (see Table 1). Finally, policy adoption event data of 30 provinces were used in this study; these data are summarized as “province-year” event history data. Following previously adopted approaches [12,18], we removed data for the years after a province established its provincial-level EMDER policy. Our period of observation for the 30 provinces was from 2008 to 2019, and statistical data are available for most provinces in this period. Ultimately, 280 “province-year” observations were analyzed in this article.

### 4.2. Measurement

Table 1 and Table 2 present the measures and descriptive statistics of all variables, respectively.

#### 4.2.1. Dependent Variable

Policy adoption was set as a dummy variable, which takes a value of ‘1’ for the years a province adopted the EMDER policy; otherwise, the value is ‘0’. Observations for a province were removed from the dataset after the policy adoption event occurred. Data for this variable were compiled from information available on the official websites of provincial governments in China. Figure 1 presents the adoption of the EMDER policy at the provincial level for each year. The unit “number of adoptions a year” represents the number of provincial governments adopting the EMDER policy in a given year.

#### 4.2.2. Independent Variables

The first set includes variables that represent characteristics that are internal to the locality. The issue salience variable was measured by the proportion of households that lived in medium high-rise dwellings within the total households of a province. The measurement of this variable is based on the following consideration. Residential dwellings in China can be divided into four types according to the number of floors: low-rise dwellings (1–3 floors), multistory dwellings (4–6 floors), medium high-rise dwellings (7–9 floors), and high-rise dwellings (10 floors and more). High-rise dwellings were equipped with elevators when they were built according to the law. Compared to residents living in multistory dwellings, residents living in medium high-rise dwellings are even more affected by the lack of an elevator. Residents in medium high-rise dwellings may more urgently advocate for changes to their physical environment because they are more aware of the physical barriers in their dwellings. The issue salience variable can be obtained only from the 2010 Population Census of China and was treated as not subject to change over time during the period 2008–2019. According to the 2010 population census, the proportion of households living in medium high-rise dwellings ranges from 0.16% (Tibet) to 16.36% (Guangdong). These figures demonstrate that different provincial governments may feel different degrees of pressure or urgency to intervene in response to public concern about elevator retrofitting in existing multifamily dwellings.

This study uses the fiscal dependency variable to reflect the vertical intergovernmental fiscal relationship. Specifically, fiscal dependency on transfer payments of a province measures the fiscal relationship between a province and the central government. Under China’s Tax-Sharing System, the extent of fiscal dependency differed considerably among provinces. In the literature [18], fiscal dependency is usually calculated by using the following equation, where ${\mathrm{FD}}_{ij}$ denotes the transfer dependency of province i in year j; ${\mathrm{Exp}}_{ij}$ and ${\mathrm{Rev}}_{ij}$ denote the budgetary expenditure and budgetary revenue, respectively, of province i in year j:

(1) $${\mathrm{FD}}_{ij}=\left({\mathrm{Exp}}_{ij}-{\mathrm{Rev}}_{ij}\right)/{\mathrm{Exp}}_{ij}$$

where ${\mathrm{Exp}}_{ij}-{\mathrm{Rev}}_{ij}$ captures the fiscal gaps of province *i* in year *j*. If ${\mathrm{Exp}}_{ij}>{\mathrm{Rev}}_{ij}$, thus indicating that province *i* has a positive fiscal deficit, then the province will rely on the fiscal transfer payments from the central government to fill the gap in financial expenditure. In contrast, if ${\mathrm{Exp}}_{ij}<{\mathrm{Rev}}_{ij}$, thus indicating that province *I* has a fiscal surplus, then province *i* does not need to rely on transfer payments from the central government to balance its budgets and has more autonomy in setting local policy priorities. This variable was obtained from the China Statistical Yearbook (2008–2019) and treated as time varying.

The second set of variables represents three diffusion mechanisms. The central signal reflects the top-down influence of the central government on provincial policy adoption; we measured the central signal as a dummy taking a value of ‘1’ for the years after the 2016 enactment of China’s first nationwide official guidelines on the construction of livable community environments for older persons; the guidelines were jointly issued by 24 central ministries. For years including and preceding 2016, the dummy takes a value of ‘0’. The city adoption variable, which represents the vertical bottom-up diffusion from local to provincial governments, was measured as the accumulated percentage of cities that had adopted the EMDER policy in a province in the previous year. The neighboring adoption variable, which represents strategic interactions among provinces, was measured as the accumulated percentage of provincial governments that adopted the EMDER policy within China in the previous year. These three variables were obtained from official government websites at the city and provincial levels.

#### 4.2.3. Control Variables

The general economic and social characteristics of provincial governments are used as control variables. We expect that population aging and the residential income of a province may increase the likelihood of the adoption of the EMDER policy by provincial governments. Population aging refers to the proportion of the population aged 65 and over to the total population of a province. Population aging reflects the level and potential challenge posed by population aging in a province. Residential income was included to control for the purchasing power of urban residents. Residential income was measured as the annual wages and salaries of local workers and staff.

### 4.3. Statistical Analysis

We tested the hypotheses on the adoption of the EMDER policy innovation across Chinese provinces by using a traditional event history analysis (EHA) model and a piecewise constant exponential (PCE) model, which is a modified EHA model. The statistical analyses strategy (EHA model) is a survival-model-based approach that has been widely used to test the drivers of the adoption of policy innovation in verified countries and domains [12,13,18]. Compared with the cross-sectional analysis, this approach can better reveal a possible causal relationship. In this study, we first use the EHA model to test the above hypotheses (see Table A1 in Appendix A). However, the EHA model assumes that the forces of change are constant over time. This assumption has been criticized as theoretically unreasonable by some scholars [18,29] and appears inconsistent with the observed dynamic diffusion process used in this study. At various stages of the EMDER policy, an obvious acceleration caused by the central signal was observed. The central signal is the 2016 national guidance that 24 central ministries jointly issued. The PCE model relatively relaxes the requirement of this assumption and allows the transition rate to vary in different periods. Thus, compared with traditional event-history-analysis (EHA) models, PCE analysis offers more appropriate modeling of the diffusion process of EMDER policy adoption [18,29]. The PCE model was employed to split the time axis into two periods (the periods before and after the 2016 national guidance), and we assume that transition rates are constant in each of these two periods but can change between the periods. Following previously adopted approaches [12,18], we removed data for the years after a province established its provincial-level EMDER policy. The data on 30 provinces from 2008 to 2019 are arranged according to 280 province-year event history observations, which were finally included in the EHA and PCE models. In the PCE model, we divided the time axis into two stages: from 2008 to 2016 and from 2017 to 2019. Finally, to obtain more reliable causality, data for all time-varying independent variables were lagged by 1 year when incorporated into the PCE models.

## 5. Results

This section presents the results of the PCE model with multiple model specifications. EHA models are used only for comparison to demonstrate the rationality of PCE models. In addition, the fitting effects of EHA models are very limited, so the results are presented only in Table A1 in the Appendix A. All time-varying independent variables were lagged for 1 year to obtain more reliable causality.

Table 3 reports the results of the PCE models on the adoption of the EMDER policies. Model 1 focuses on the effects of internal determinants on provincial policy adoptions while assuming that no diffusion mechanisms are at work. The results show that policy adoption by provincial governments in China can be explained by various factors internal to the locality. First, issue salience, which indicates the potential degree of pressure being applied by the target population, significantly affects the likelihood that a provincial government adopts the EMDER policy. Second, fiscal dependency is estimated to increase the likelihood of policy adoption. Provinces that are more financially dependent on the central government are also more likely to adopt the policy. Third, provinces with a higher level of population aging and more affluent residents (residential consumption level) are more likely to adopt the policy.

Next, Models 2–5 examine the simultaneous effects of both internal determinants and external diffusion mechanisms on policy adoption by provinces. The effects of some factors have evidently changed. In Model 2, the top-down signal from the central government strongly affects the EMDER policy adoption by provincial governments. This result indicates that provincial governments were more likely to formulate the EMDER policy after the release of the Guidelines on the Construction of a Livable Environment for Older Adults, which were jointly issued by 24 central ministries in 2016. However, two internal characteristics (issue salience and population aging) remain insignificant.

Model 3 examines the effects of the combination of two vertical diffusion mechanisms (central policy signal and city adoption). Surprisingly, negative coefficients on the city adoption variable support the “Pressure Value Effect Hypothesis” instead of the “Snowball Effect Hypothesis”, thereby suggesting that the increasing adoption of the EMDER policies for older people by city governments within one province will decrease the likelihood of provincial policy adoption. However, the top-down signal from the central government remains insignificant.

Model 4 examines the effects of the combined top-down vertical diffusion mechanism and horizontal diffusion mechanism. Positive coefficients on neighboring adoptions support the horizontal province-to-province diffusion of policy innovation, thereby implying that provincial government officials formulate the EMDER policy under the influence of neighboring provinces throughout China.

In Model 5, the effects of all covariates are further simultaneously analyzed to obtain a more rigorous result. From Models 1 to 5, the most obvious change was that the influence of multiple internal determinants decreased significantly after the diffusion mechanisms were included. For internal determinants, the effect of issue salience is no longer significant. However, the two diffusion mechanisms of city adoption and neighboring adoption were still estimated to have negative and positive effects, respectively. These results may indicate that the adoption of EMDER policy innovation by Chinese provincial governments was based more on institutional factors than rational calculations based solely on local characteristics.

## 6. Discussion

The topic of this study is highly relevant, as the population aging in many countries reveals that the housing stock is not sufficiently designed to meet the needs of older people with lower functional capacity, and there is thus a high demand for HAs [1,8,10]. Most existing studies focus mainly on housing accessibility problems and their health outcomes for older adults [3,30,31]. However, very few studies have explored policy innovation on HAs for older adults from the perspective of policy processes [6]. Actually, population aging and the changing needs of older adults require considerable service innovation and policy innovation to support the daily lives of older people. Taking the EMDER policy as an example, this study attempts to investigate why provincial governments adopt policy innovations on major HA for older adults in China. This study may contribute to the understanding of housing well-being for older adults internationally.

First, most existing studies pay attention to the minor HA items; however, this study highlights the significant challenges of major HAs (elevator retrofitting) in China. Minor HAs refer to projects with relatively low cost and difficulty and are usually located in the indoor environment; examples of minor HAs include grab bars and wheelchair accommodation [4]. Actually, studies on minor HA items and their economic or health outcomes are in the mainstream of existing research [3,30]. Recently, many studies have reported that major HAs, especially elevator retrofitting, more greatly affect the daily life and health well-being of older people [4,6]. However, the study takes China’s EMDER policy as an example and finds that the delivery of major HAs is more complex than that of minor HAs. Major HAs are more costly, they easily trigger neighborhood conflicts, and they encounter the “not in my back yard” phenomenon. Consequently, major HAs are beyond the capability of the vast majority of older adults and their families. This study advocates that major HAs should be given higher policy priority and promoted by the adoption of policy innovations by governments at multiple levels.

Second, existing studies mainly explore the role of city or municipal governments; however, this study shows the relevance of superior provincial governments in promoting major HAs for older adults. City or municipal governments have traditionally been responsible for housing provision and adaptation for residents. However, because the delivery of major HAs is costly and complex, a large gap exists between cities or municipalities in their ability to provide major HAs (elevator retrofitting). Municipalities and cities with fewer resources and decreasing population levels have been identified as having the greatest difficulties in providing accessible housing for older adults [6,7,10]. Policy coordination and planning at superior provincial governments are relevant to improving older adults’ access to major HA services. However, this study identifies the significant “pressure valve effect” on EMDER policy adoption between provincial and city governments. That is, the increasing adoption of major HA policies for older people by city governments within one province will decrease the likelihood of provincial policy adoption. This finding indicates that housing accessibility problems faced by older Chinese people are still perceived as a local matter for the subordinate city government. A possible explanation is that the increasing adoption of EMDER policies by subordinate cities within a province may make this policy problem less salient and reduce the potential pressure faced by superior provincial governments [6,7,10]. This explanation indicates that if we want to improve the housing well-being of older Chinese people on a larger scale and in a sustainable way, policy coordination and planning at the provincial level need to be strengthened, and the adoption of policy innovation by provincial governments should be encouraged.

Third, the positive coefficient of the neighboring adoption variable indicates that the quest for legitimacy may promote the adoption of policy innovation on major HAs by Chinese provincial governments. Respect for older senior citizens is a traditional virtue in China. Therefore, policy innovations to improve the housing well-being of older people are consistent with social norms and culture and are more likely to gain legitimacy. The increasing adoption of the EMDER policy by neighboring provincial governments may further strengthen the legitimacy of this policy and thus create legitimacy pressure on other nonadopters and inspire the adoption of isomorphic policies by provincial governments.

Last, the positive coefficients of the financial dependency variable demonstrate that fiscal transfer payments from the central government could significantly influence some provincial governments’ policy decisions on the adoption of the EMDER policy. These transfer payments have become a significant component of local expenditure and are particularly important to provinces with poor tax bases. China’s central government attaches great importance to the welfare of older adults and has formulated several national development plans to actively respond to population aging since 2000. Thus, provincial governments, which are more financially dependent on the central government, have greater internal incentives to comply with the central government’s policy goals. Thus, fiscal dependency on the central government significantly facilitates the provincial government’s adoption of major HA policy innovations in China.

The study also has a few limitations. First, this study measures only whether the central government sends a policy signal on HA for older adults. Future studies should take further steps to measure the intensity of policy intervention from the central government. Second, issue salience is measured as a time-constant variable collected from the 2010 Population Census of China, but this variable can be measured as a time-varying variable in future studies by developing new data sources.

## 7. Conclusions

Major HAs for older adults are highly relevant. This study highlights the significant challenges of major HAs, such as elevator retrofitting in existing multifamily dwellings, including the costly, difficult, and easily encountered “not in my back yard” phenomenon. Then, the study explores the factors that facilitate provincial governments’ adoption of major HA policy innovation that benefits primarily older adults in China. Specifically, the pressure valve effect of city adoption reminds us that if we want to address this pressing problem on a larger scale and in a sustainable way, provincial governments’ adoption of major HA policies should be given a higher priority. In addition, other significant factors include neighboring adoption and financial dependency. In conclusion, the central government can promote provincial governments’ adoption of major HA policy innovation by enhancing the legitimacy of such policy or by using fiscal transfer payments.

## Figures and Tables

**Table 1:** Measurements of Variables.

Variables	Description of Measurements	Data Source
Policy adoption	Whether a provincial government adopts the EMDER policy in the current year (1) or not (0).	Collected by author
Issue salience	The proportion of households living in medium high-rise dwellings within the total number of households of a province in the year 2000.	Population Census of China 2010
Fiscal dependency	The fiscal dependency of province *i* in year *j* is calculated as: (budgetary expenditure *_ij_*—budgetary revenue *_ij_*)/budgetary expenditure *_ij_*	China Statistical Yearbook (2008–2019)
Central policy signal	Before (0) or during and after the year 2016 (1), when the 24 central ministries jointly promulgated national guidance to promote age-friendly cities and communities.	Collected by author
City adoption	Cumulative percentage of cities that had adopted the EMDER policy of a province in the previous year.	Author’s calculation
Neighboring adoption	Cumulative percentage of provinces that had adopted the EMDER policy of China in the previous year.	Author’s calculation
Population aging	The proportion of the population aged 65 and over to the total population of a province in the previous year	China Statistical Yearbook (2008–2019)
Consumption level	Consumption expenditure of urban residents of a province in the previous year.	

**Table 2:** Descriptive statistics of variables.

Variables	2008–2019			2008–2016			2017–2019		
	N	Mean	SD	N	Mean	SD	N	Mean	SD
Policy adoption	280	16.00	5.71	225	6.00	2.67	55	10.00	18.18
Issue salience	280	0.05	0.04	225	0.05	0.04	55	0.04	0.04
Fiscal dependency	280	0.56	0.17	225	0.55	0.17	55	0.59	0.15
Neighboring adoption	280	0.15	0.12	225	0.10	0.06	55	0.35	0.10
Central policy signal	280	55.00	19.64	225	0.00	0.00	55	55.00	100.00
City adoption	280	0.02	0.08	225	0.00	0.02	55	0.09	0.16
Population aging	280	0.09	0.02	225	0.09	0.02	55	0.10	0.02
Consumption level	280	18,039.61	6818.05	225	16,087.87	5358.19	55	26,024.04	6346.57

**Table 3:** Piecewise constant exponential models for EMDER policy adoption by provincial governments in China.

	Model 1	Model 2	Model 3	Model 4	Model 5
	*β* (se)	*β* (se)	*β* (se)	*β* (se)	*β* (se)
Central policy signal		4.11 (1.16) ***	2.08 (1.25)	2.36 (1.61)	0.22 (1.31)
City adoption			−7.84 (2.96) **		−8.26 (2.62) **
Neighboring adoption				6.37 (3.32) *	7.86 (2.92) *
Issue salience	11.69 (5.78) *	9.50 (5.90)	7.61 (5.80)	11.15 (5.87)	8.31 (5.69)
Financial dependency	12.38 (2.28) ***	6.20 (2.69) *	7.47 (2.50) **	5.33 (2.77) *	5.58 (2.49) *
Population aging	53.91 (26.27) *	25.98 (20.98)	42.65 (21.68) *	21.55 (18.93)	44.32 (18.51) *
Consumption level	0.0003 (0.00004) ***	0.0002 (0.00005) **	0.0003 (0.00006) ***	0.0001 (0.00005) *	0.00002 (0.00005) ***
_cons	−24.52 (3.04) ***	−17.06 (3.03) ***	−20.53 (3.07) ***	−15.94 (3.01) ***	−19.21 (2.75) ***
N	280	280	280	280	280
Log-likelihood	35.46	41.36	45.33	43.26	48.85
LR chi-squared	119.68	131.49	139.42	135.28	146.46

The convention is *** *p* < 0.001, ** *p* < 0.05, and * *p* < 0.10.

## Data Availability

Not applicable.

## Appendix A

**Table A1:** Event history analysis (EHA) models for EMDER policy adoption by provincial governments in China.

	Model 1	Model 2	Model 3	Model 4	Model 5
	*β* (se)	*β* (se)	*β* (se)	*β* (se)	*β* (se)
Central policy signal		3.23 (none)	2.37 (none)	3.16 (none)	3.37 (none)
City adoption			−1.56 (2.70)		−1.56(2.70) *
Neighboring adoption				2.09 (none)	1.96 (none)
Issue salience	9.29 (5.86)	9.29 (5.86)	9.09 (5.76)	9.29 (5.86)	9.09 (5.76)
Financial dependency	−1.20 (2.89)	−1.20 (2.89)	−1.23 (2.87)	−1.2 (2.89)	−1.23 (2.87)
Population aging	9.60 (17.81)	9.60 (17.81)	11.08 (17.60)	9.60 (17.81)	11.08 (17.60)
Consumption level	−0.00004 (0.00007)	0.00004 (0.00007)	0.−00002 (0.00008)	−0.00004 (0.00007)	−0.00002 (0.00008)
N	280	280	280	280	280
Log-likelihood	−45.84	−45.84	−45.66	−45.84	−45.66
LR chi-squared	4.65	4.65	5.00	4.65	5.00

The convention is *** *p* < 0.001, ** *p* < 0.05, and * *p* < 0.10.
